# Supplementary material for: Loss of spatacsin impairs cholesterol trafficking and calcium homeostasis
Source: Commun Biol. 2019 Oct 17;2:380. doi: 10.1038/s42003-019-0615-z (PMC6797781; doi:10.1038/s42003-019-0615-z)
Supplement: Supplementary file 1 — Supplementary Information [file 42003_2019_615_MOESM1_ESM.pdf]

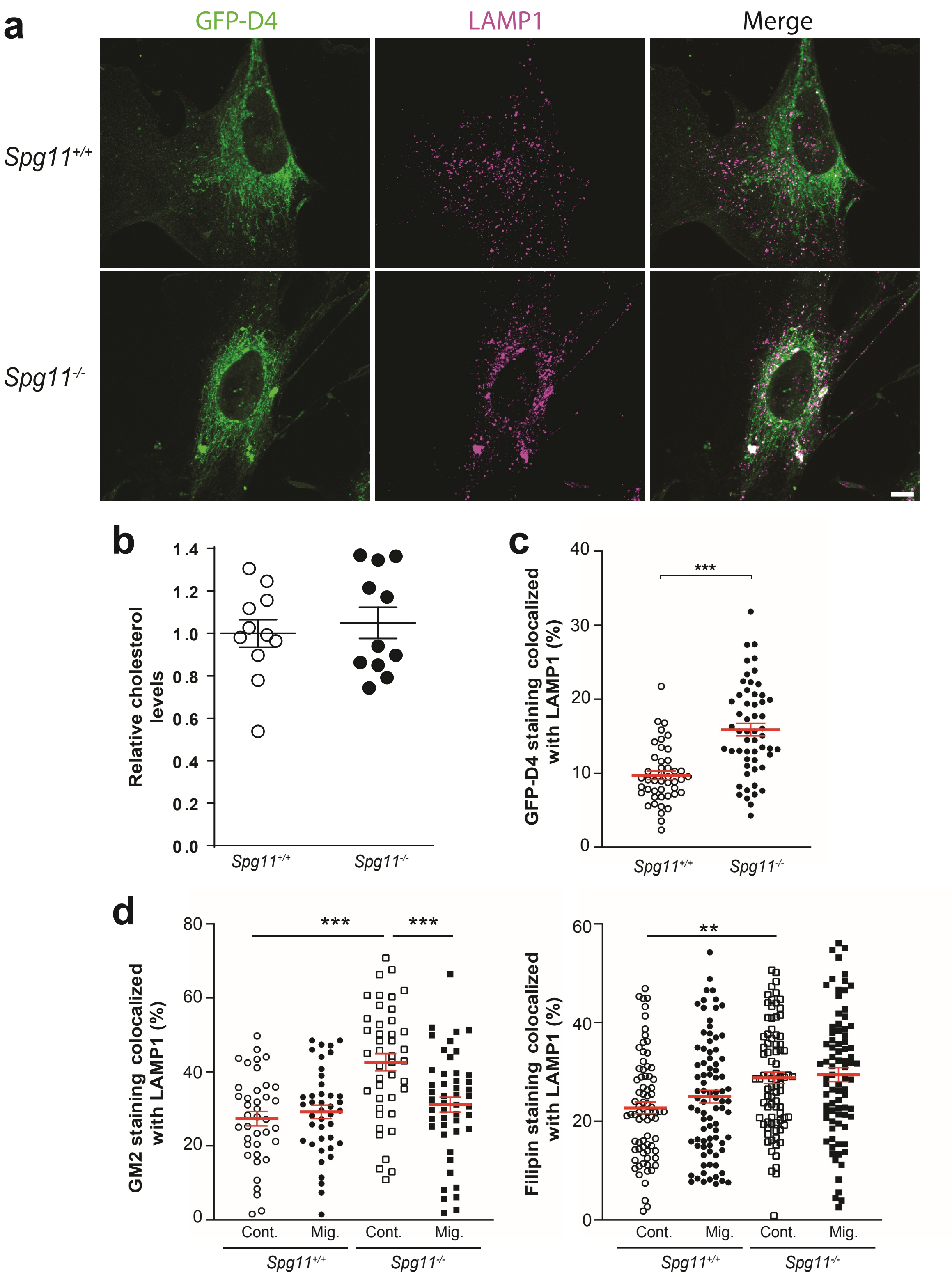

Supplementary Figure 1. The loss of spatacsin promotes the accumulation of cholesterol in late endosomes/lysosomes. a. Staining of cholesterol with the probe GFP-D4 and late endosomes/lysosomes by the marker LAMP1 in *Spg11*$^{+/+}$ and *Spg11*$^{-/-}$ fibroblasts. Scale bar: 10 µm. **b.** Biochemical quantification of total cholesterol levels in *Spg11*$^{+/+}$ and *Spg11*$^{-/-}$ fibroblasts. N = 11 in six independent experiments. Mann-Whitney test: p = 0.86. **c.** Quantification of the amount of GFP-D4 staining colocalized with the marker LAMP1, showing a higher amount of cholesterol in late endosomes/lysosomes in *Spg11*$^{+/+}$ and *Spg11*$^{-/-}$ fibroblasts. The graph shows the mean ± SEM. N > 45 cells analyzed in three independent experiments. T-test: ***p < 0.0001. **d.** Quantification of the amount of GM2 (left panel) and filipin (right panel) staining colocalized with the marker LAMP1 in *Spg11*$^{+/+}$ and *Spg11*$^{-/-}$ neurons that were treated with miglustat (Mig. 100 µM) to prevent accumulation of gangliosides. Miglustat treatment prevents the accumulation of GM2 ganglioside colocalized with LAMP1 in *Spg11*$^{-/-}$ neurons, but did not modify the levels of cholesterol staining (filipin) colocalized with the LAMP1 marker in *Spg11*$^{-/-}$ neurons. The graph shows the mean ± SEM. N > 75 cells from three independent experiments. One way ANOVA: *** p<0.001; ** p<0.01.

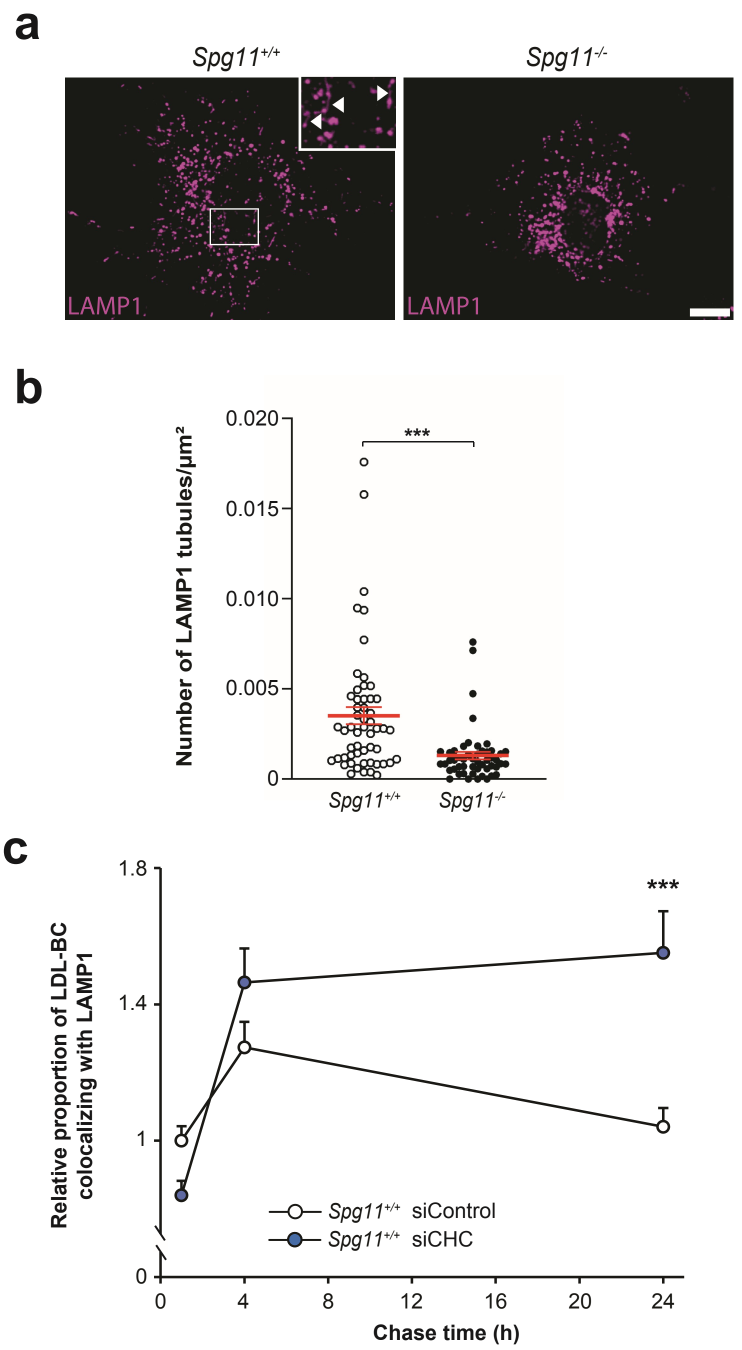

Supplementary Figure 2. The formation of tubules on lysosomes contributes to the clearance of cholesterol. **a.** Live imaging of *Spg11*$^{+/+}$ and *Spg11*$^{-/-}$ fibroblasts transfected with a vector expressing LAMP1 fused to mCherry. Note the presence of tubules (arrowheads in insets) emanating from late endosomes/lysosomes in *Spg11*$^{+/+}$ fibroblasts. Scale bar: 10 µm. **b.** Quantification of the number of LAMP1-positive tubules in *Spg11*$^{+/+}$ and *Spg11*$^{-/-}$ fibroblasts. The graph shows the mean ± SEM. N > 200 cells analyzed in three independent experiments. T-test: ***p < 0.0001. **c.** Quantification of the amount of Top-Fluor cholesterol colocalized with the LAMP1 marker in control fibroblasts transfected with control siRNA or siRNA downregulating CHC. Fibroblasts were incubated with LDL loaded with Top-Fluor cholesterol for 2 h, rinsed, and analyzed after various chase times. The graph shows the mean ± SEM. N > 45 cells analyzed in three independent experiments. Two-way ANOVA followed by Holm-Sidak multiple comparison test: *** p < 0.0001.

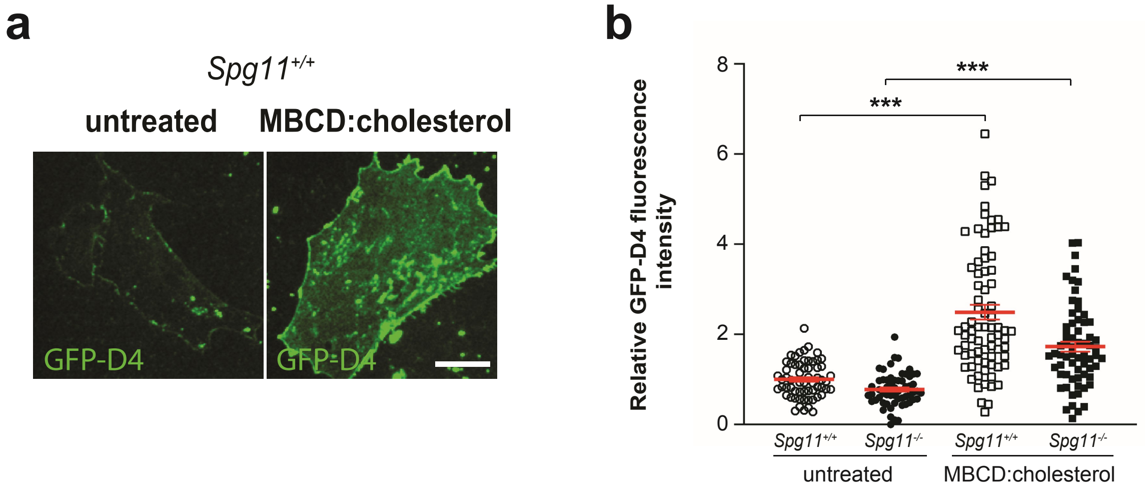

Supplementary Figure 3. Loading of the plasma membrane with cholesterol. a. Live imaging of plasma-membrane cholesterol in cells stained with the probe GFP-D4, showing that loading of fibroblasts with methyl-β-cyclodextrin (MBCD) coupled with cholesterol increases the amount of plasma membrane cholesterol. Scale bar: 10 µm. **b.** Relative amount of plasma membrane cholesterol monitored by staining with the probe GFP-D4 in live cells. The graph shows the mean ± SEM. N > 55 cells analyzed in three independent experiments. Two-way ANOVA followed by the Holm-Sidak multiple comparison test: ***$p < 0.0001$.

Boutry et al. Supplementary Figure 4

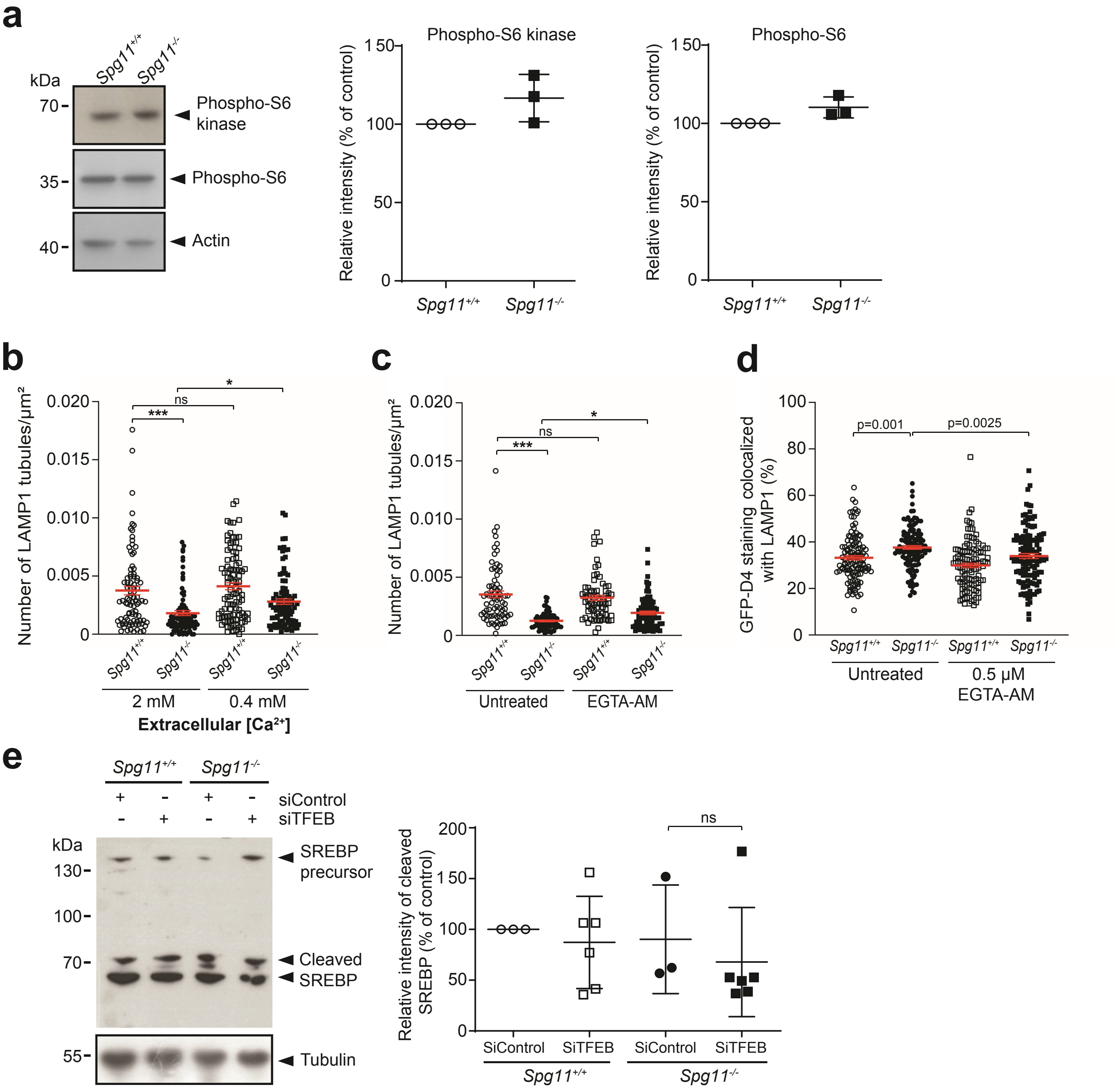

Supplementary Figure 4. Decreasing the extracellular calcium concentration restores the formation of tubules and normal cholesterol levels in lysosomes in *Spg11*$^{-/-}$ fibroblasts. a. Western blots showing the amount of phosphorylated S6 kinase and S6 protein, as well as actin as a loading control in *Spg11*$^{+/+}$ and *Spg11*$^{-/-}$ fibroblasts. Left panel: quantification of the amount of phosphorylated S6 kinase and S6 normalized to actin. The graphs show the mean ± SEM. **b.** Quantification of the number of LAMP1-positive tubules in *Spg11*$^{+/+}$ and *Spg11*$^{-/-}$ fibroblasts expressing LAMP1-mCherry, analyzed by live imaging. The number of LAMP1-positive tubules is lower in *Spg11*$^{-/-}$ than *Spg11*$^{+/+}$ fibroblasts in a medium containing 2 mM $CaCl_2$. The number of tubules in *Spg11*$^{-/-}$ fibroblasts increases when they are incubated in a medium containing 0.4 mM $CaCl_2$ for 1 h. The graph shows the mean ± SEM. N > 45 cells analyzed in three independent experiments. Two-way ANOVA followed by the Holm-Sidak multiple comparison test: *p = 0.042, ***p < 0.0001. **c.** Quantification of the number of LAMP1-positive tubules in *Spg11*$^{+/+}$ and *Spg11*$^{-/-}$ fibroblasts expressing LAMP1-mCherry. The number of tubules increases in *Spg11*$^{-/-}$ fibroblasts when they are incubated in a medium containing 0.5 µM EGTA-AM for 1 h. The graphs show the mean ± SEM. N > 60 cells analyzed in four independent experiments. Two-way ANOVA followed by the Holm-Sidak multiple comparison test: *p = 0.030, ***p < 0.0001. **d.** Incubation of neurons in a medium containing 0.5µM EGTA-AM for 1 h decreases

the amount of cholesterol colocalized with a late endosome/lysosome marker in *Spg11*$^{-/-}$ neurons. The graph represents the mean ± SEM. N > 110 cells analyzed in at least three independent experiments. Two-way ANOVA followed by the Holm-Sidak multiple comparison test. **e.** Western blot showing the levels of SREBP precursor and cleaved form in *Spg11*$^{+/+}$ and *Spg11*$^{-/-}$ fibroblasts transfected with a control siRNA or a siRNA downregulating TFEB. The cleaved form of SREBP allows it transcriptional activity, and the expression of enzymes mediating cholesterol synthesis. Left panel: quantification of the amount of cleaved form of SREBP normalized to tubulin. The graph shows the mean ± SEM (3 independent experiments).

Boutry et al. Supplementary Figure 5

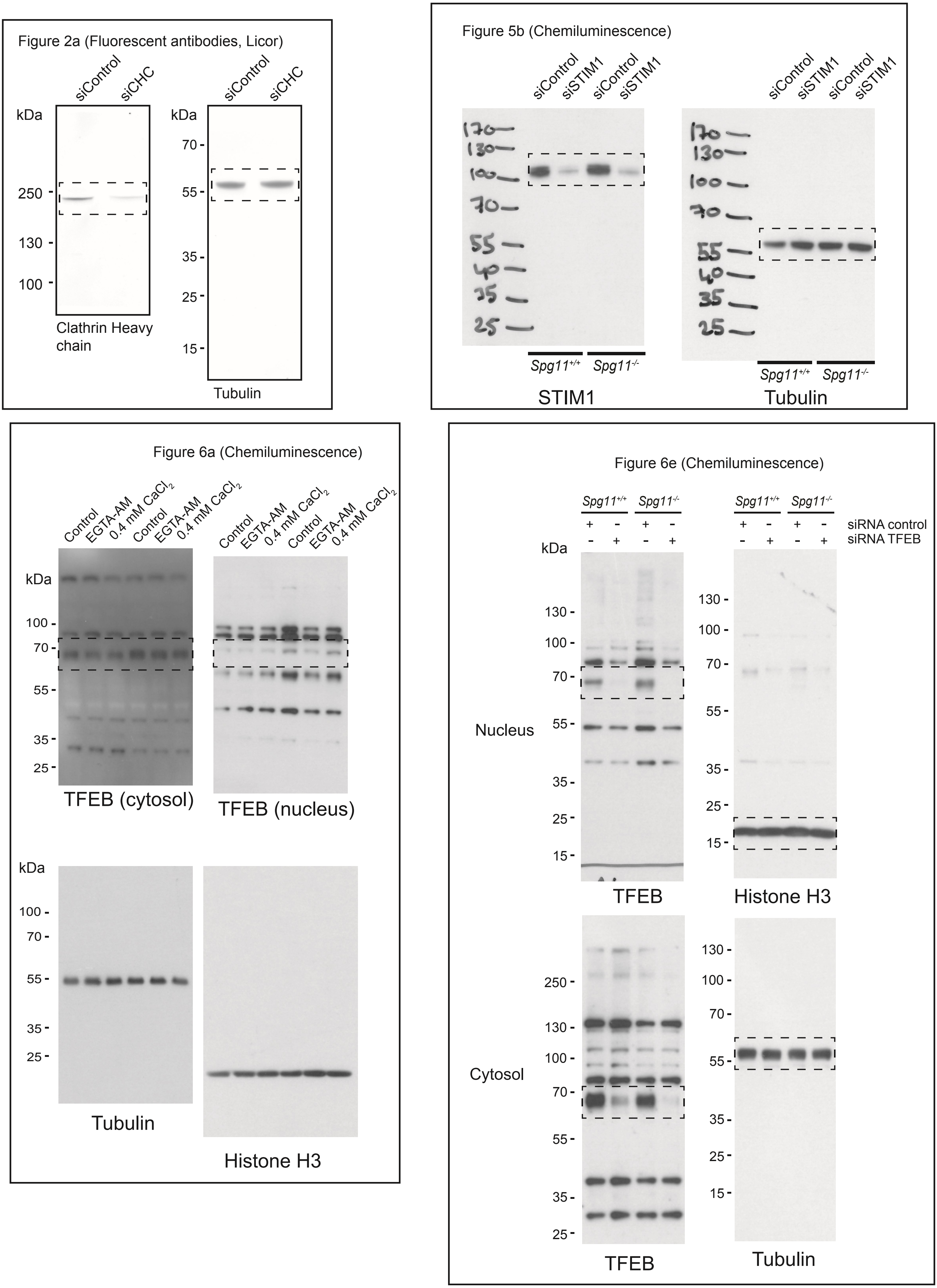

Boutry et al. Supplementary Figure 5 (continued)

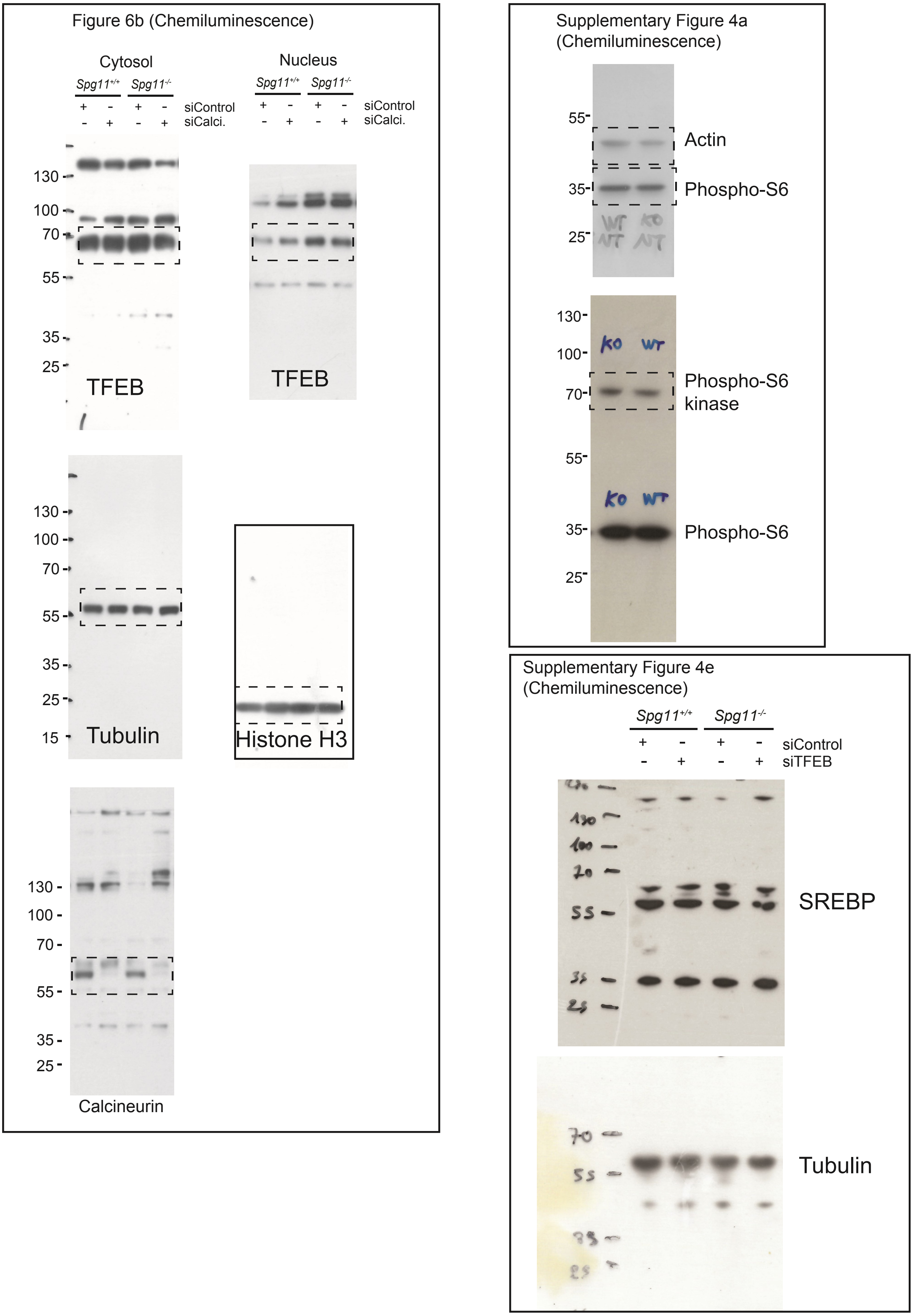

Supplementary Figure 5. Uncropped images of western blots used in Figures 2, 5 and 6 as well as Supplementary figure 4.
