# Supplementary material for: Loss of spatacsin impairs cholesterol trafficking and calcium homeostasis
Source: Commun Biol. 2019 Oct 17;2:380. doi: 10.1038/s42003-019-0615-z (PMC6797781; doi:10.1038/s42003-019-0615-z)
Supplement: Supplementary file 2 — Description of Additional Supplementary Files [file 42003_2019_615_MOESM2_ESM.docx]

**Description of Additional Supplementary Files**

**File Name** : Supplementary Data 1

**Description** :This file contains all data underlying the graphs presented in the figures.
