# Supplementary material for: Loss of spatacsin impairs cholesterol trafficking and calcium homeostasis
Source: Commun Biol. 2019 Oct 17;2:380. doi: 10.1038/s42003-019-0615-z (PMC6797781; doi:10.1038/s42003-019-0615-z)
Supplement: Supplementary file 4 — Reporting Summary [file 42003_2019_615_MOESM4_ESM.pdf]

nature research

Corresponding author(s): Frédéric Darios

Last updated by author(s): Jul 22, 2019

Reporting Summary

Nature Research wishes to improve the reproducibility of the work that we publish. This form provides structure for consistency and transparency in reporting. For further information on Nature Research policies, see Authors & Referees and the Editorial Policy Checklist.

Statistics

For all statistical analyses, confirm that the following items are present in the figure legend, table legend, main text, or Methods section.

n/a	Confirmed	
☐	☒	The exact sample size (n) for each experimental group/condition, given as a discrete number and unit of measurement
☐	☒	A statement on whether measurements were taken from distinct samples or whether the same sample was measured repeatedly
☐	☒	The statistical test(s) used AND whether they are one- or two-sided<br>*Only common tests should be described solely by name; describe more complex techniques in the Methods section.*
☐	☒	A description of all covariates tested
☐	☒	A description of any assumptions or corrections, such as tests of normality and adjustment for multiple comparisons
☐	☒	A full description of the statistical parameters including central tendency (e.g. means) or other basic estimates (e.g. regression coefficient) AND variation (e.g. standard deviation) or associated estimates of uncertainty (e.g. confidence intervals)
☐	☒	For null hypothesis testing, the test statistic (e.g. F, t, r) with confidence intervals, effect sizes, degrees of freedom and P value noted<br>*Give P values as exact values whenever suitable.*
☒	☐	For Bayesian analysis, information on the choice of priors and Markov chain Monte Carlo settings
☒	☐	For hierarchical and complex designs, identification of the appropriate level for tests and full reporting of outcomes
☒	☐	Estimates of effect sizes (e.g. Cohen's d, Pearson's r), indicating how they were calculated

Our web collection on statistics for biologists contains articles on many of the points above.

Software and code

Policy information about availability of computer code

Data collection	Zen2, Metamorph, HCImage 4
Data analysis	ImageJ, Matlab, HCImage 4, GraphPad Prism 6

For manuscripts utilizing custom algorithms or software that are central to the research but not yet described in published literature, software must be made available to editors/reviewers. We strongly encourage code deposition in a community repository (e.g. GitHub). See the Nature Research guidelines for submitting code & software for further information.

Data

Policy information about availability of data

All manuscripts must include a data availability statement. This statement should provide the following information, where applicable:

- Accession codes, unique identifiers, or web links for publicly available datasets
- A list of figures that have associated raw data
- A description of any restrictions on data availability

Raw data are available from the corresponding author upon reasonable request

Field-specific reporting

Please select the one below that is the best fit for your research. If you are not sure, read the appropriate sections before making your selection.

☒ Life sciences ☐ Behavioural & social sciences ☐ Ecological, evolutionary & environmental sciences

For a reference copy of the document with all sections, see nature.com/documents/nr-reporting-summary-flat.pdf

Life sciences study design

All studies must disclose on these points even when the disclosure is negative.

Sample size	No calculation was made to determine sample size
Data exclusions	No data were excluded
Replication	All experiment were repeated at leat three time independently, using mouse embryonic fibroblasts obtained from independent mouse breeding
Randomization	N/A
Blinding	No blinding was used

Reporting for specific materials, systems and methods

We require information from authors about some types of materials, experimental systems and methods used in many studies. Here, indicate whether each material, system or method listed is relevant to your study. If you are not sure if a list item applies to your research, read the appropriate section before selecting a response.

Materials & experimental systems

n/a	Involved in the study	
☐	☒	Antibodies
☒	☐	Eukaryotic cell lines
☒	☐	Palaeontology
☐	☒	Animals and other organisms
☒	☐	Human research participants
☒	☐	Clinical data

Methods

n/a	Involved in the study	
☒	☐	ChIP-seq
☒	☐	Flow cytometry
☒	☐	MRI-based neuroimaging

Antibodies

Antibodies used	Mouse anti alpha-tubulin, clone DM1A, Abcam, ab7291<br>rat anti LAMP1, Development Studies Hybridoma Bank, clone 1D4B<br>mouse anti Clathrin heavy chain, clone 23, BD Biosciences, 610499<br>rabbit anti Stim1, clone D88E10, Cell Signaling, 5668<br>rabbit anti TFEB, ProteinTech, 13372-1-AP<br>rabbit anti histone H3, Cell Signaling, 4499<br>Rabbit anti PPP3CB (calcineurin), Abcam ab191374<br>Rabbit ant SREBP, Abcam ab30682
Validation	Validation in Fig 2A (Clathrin heavy chain), Fig 5B (Stim1), Fig 6D (TFEB), Fig 6F (PPP3CB)<br>Mouse anti alpha-tubulin, rat anti LAMP1 and rabbit anti histone H3 are routinely used in many studies

Animals and other organisms

Policy information about studies involving animals; ARRIVE guidelines recommended for reporting animal research

Laboratory animals	Male and female Spg11+/- C57Bl6/N (described in Branchu et al, 2017) were bread in the animal facility of the host institute
Wild animals	N/A
Field-collected samples	N/A
Ethics oversight	Ministère de l'Agriculuture, APAFIS-5199

Note that full information on the approval of the study protocol must also be provided in the manuscript.
